# A pharmacokinetic study of lipegfilgrastim in children with Ewing family of tumors or rhabdomyosarcoma

**DOI:** 10.1007/s00280-016-3216-2

**Published:** 2016-12-16

**Authors:** Margarita B. Belogurova, Zoryana P. Kizyma, Miklós Garami, Mónika Csóka, Michael J. Lamson, Anton Buchner, Peter Bias, Andreas Lammerich

**Affiliations:** 1 Pediatric Oncology and Hematology, City Clinical Hospital #31, 3 Dinamo Pr., St. Petersburg, Russian Federation 197110; 2 Department of Surgery, West Ukrainian Specialized Children’s Medical Center, Lviv, Ukraine; 3 Unit of Solid Tumors, 2nd Department of Pediatrics, Semmelweis University, Budapest, Hungary; 4 Nuventra, Inc, Durham, NC USA; 5 Teva ratiopharm, Merckle GmbH, Ulm, Germany

**Keywords:** Chemotherapy, Pediatric, G-CSF, Neutropenia, Phase 1, Pharmacodynamics

## Abstract

**Purpose:**

Neutropenia is a common complication from chemotherapy, limiting optimal dosing and treatment. Lipegfilgrastim is a long-acting granulocyte colony-stimulating factor developed for the management of chemotherapy-induced neutropenia. The objectives of this phase 1, multinational, open-label, single-arm study were to characterize the pharmacokinetics (PK) and pharmacodynamics (PD) of a single body weight-adjusted dose of lipegfilgrastim and to evaluate the efficacy, safety, and tolerability of the drug in children with Ewing family of tumors or rhabdomyosarcoma treated with myelosuppressive chemotherapy.

**Methods:**

Enrolled patients received lipegfilgrastim (100 µg/kg) 24 h after the last chemotherapy treatment in week 1. Patients were stratified into three age groups: 2 to <6, 6 to <12, and 12 to <18 years. Blood samples for PK analyses were obtained at baseline and at 3, 8, 24, 30, 48, 72, 96, 144, and 240 h postdose for the two oldest groups and up to 144 h in the youngest group.

**Results:**

Twenty-one patients were enrolled and received lipegfilgrastim, seven in each age group. Lipegfilgrastim exposure levels were comparable across age groups, with concentrations maintained over a prolonged period after a single injection. Differences in PD were mainly associated with chemotherapy type. Most investigator-reported adverse events were attributed to chemotherapy and not to lipegfilgrastim. Severe adverse events were noted in 57% of patients; febrile neutropenia, leukopenia, neutropenia, and thrombocytopenia were more frequent among the oldest patients.

**Conclusions:**

Results support the use of a body weight-adjusted dose to achieve equivalent initial peak exposure levels of lipegfilgrastim in children of various ages.

**Electronic supplementary material:**

The online version of this article (doi:10.1007/s00280-016-3216-2) contains supplementary material, which is available to authorized users.

## Introduction

Dose-intensive chemotherapy provides substantial clinical benefit for pediatric patients with nonmetastatic sarcomas, as shown by studies indicating that the intensity of chemotherapy is correlated with improved outcomes [1]. However, chemotherapy-induced neutropenia and associated infection-related complications may limit optimal dosing [2, 3]. Thus, decreasing the severity or shortening the duration of neutropenia following chemotherapy may facilitate administration of intensive chemotherapy and improve efficacy and safety.

The definitions of neutropenia and the associated risk factors among children are consistent with those in the adult population [4]. Granulocyte colony-stimulating factor (G-CSF) is routinely administered to adults and children to counter dose-limiting effects of chemotherapy. Filgrastim is currently approved for use in children as a daily injection for up to 14 days [5]. However, the drug’s short half-life (~3.5 h) is due primarily to rapid clearance by renal excretion, necessitating a daily injection that may be problematic for children. Pegfilgrastim (Neulasta, Amgen Inc., USA), a human recombinant G-CSF conjugated at the N terminus to a single 20-kDa polyethylene glycol (PEG) molecule, is administered to adults as a single injection after each cycle of cytotoxic chemotherapy but has not been approved for children [6]. Prospective, randomized studies have shown that the efficacy and safety of pegfilgrastim are similar to those of filgrastim among pediatric patients with sarcoma, without evidence for differences in the pharmacologic properties between adults and children [7–10].

Lipegfilgrastim (Lonquex, Merckle Biotec GmbH, Germany, Teva Pharmaceuticals, Israel) is comparable to pegfilgrastim. It is a long-acting form of filgrastim comprised of a single PEG molecule conjugated to filgrastim at the natural G-CSF glycosylation site via a carbohydrate linker. It has been proposed that lipegfilgrastim and pegfilgrastim share the same elimination pathway by neutrophil-mediated clearance, which is saturated at higher doses [11]. However, results from a recent preclinical study suggested that lipegfilgrastim is more resistant to degradation and retains greater functional activity than pegfilgrastim following exposure to purified human neutrophil elastase or isolated human neutrophils [12]. Consistent with a self-regulating clearance mechanism, serum lipegfilgrastim concentrations decline slowly during the initial neutropenic phase following chemotherapy and decline rapidly as the neutrophil pool recovers [13].

Lipegfilgrastim is approved by the European Medicines Agency for reducing the duration of neutropenia and the incidence of febrile neutropenia in adult patients treated with cytotoxic chemotherapy; this approval was based on a randomized phase 3, active-controlled study with breast cancer patients [14] and a randomized phase 3, placebo-controlled study with lung cancer patients [15]. In two studies conducted in adult patients with breast cancer, mean peak concentrations (*C*_max_) of 227 and 266 ng/mL were achieved with median time-to-peak concentration (*t*_max_) values of 44 and 48 h, respectively, following a single 6-mg subcutaneous dose of lipegfilgrastim during cycle 1 of chemotherapy with doxorubicin and docetaxel. In a separate study in adult patients with non-small cell lung cancer, a similar lipegfilgrastim treatment during chemotherapy with cisplatin and etoposide resulted in a mean *C*_max_ of 317 ng/mL and median *t*_max_ of 24 h [13].

There is no evidence from clinical studies of any differences in pharmacologic properties and mechanism of action of pegfilgrastim between adult and pediatric populations [7–10]. Because dosing is adjusted according to body weight, lipegfilgrastim, with a molecular composition resembling that of pegfilgrastim, is expected to achieve peak exposure levels and to display a pharmacologic profile in children similar to that previously observed in adults.

The objectives of this study were to characterize the pharmacokinetics (PK) and pharmacodynamics (PD) of a single body weight-adjusted dose (100 µg/kg) of lipegfilgrastim administered as a subcutaneous injection and to evaluate the efficacy, safety, and tolerability of the drug in children with Ewing family of tumors or rhabdomyosarcoma treated with myelosuppressive chemotherapy.

## Materials and methods

### Study design

This phase 1, multinational open-label, nonrandomized study included a screening period and a 3-week treatment and assessment period. Patients were stratified into three age groups: 2 to <6 years, 6 to <12 years, and 12 to <18 years. Enrollment of patients 2 to <6 years of age did not begin until PD and safety data from the two older groups were available and reviewed by an independent data monitoring committee. The planned sample size of 21 patients was considered sufficient to allow exploratory analysis.

The study was conducted in accordance with the International Conference on Harmonisation, Good Clinical Practice Consolidated Guideline (E6), any applicable national and local laws and regulations, and the Declaration of Helsinki. Written informed consent from parents or legal guardians of each patient and assent from adolescent patients were obtained prior to study entry. This trial was registered at www.clinicaltrials.gov (NCT01585649).

### Patients

Eligible patients were children 2 to <18 years of age with Ewing family of tumors or rhabdomyosarcoma scheduled to receive myelosuppressive chemotherapy. Regimens comprised vincristine/ifosfamide/doxorubicin/etoposide (VIDE) or vincristine, doxorubicin, and cyclophosphamide alternating with ifosfamide and etoposide (VDC/IE) for patients with Ewing family of tumors or vincristine/actinomycin D/cyclophosphamide (VAC), VDC/IE, or ifosfamide/vincristine/actinomycin D (IVA) for patients with rhabdomyosarcoma. Chemotherapy dosages and schedules are shown in Table S1.

Inclusion criteria were generally based on those used in studies with adults, with the addition of minimum body weight to ensure that sufficient blood volume would be drawn, in accordance with recommendations by the European Medicines Agency related to clinical trials in the pediatric population [16]. Other inclusion criteria were body weight ≥15 kg for patients 6 to <18 years of age and ≥12.5 kg for patients 2 to <6 years of age, white blood cell count >2.5 × 10^9^/L, absolute neutrophil count (ANC) ≥1.5 × 10^9^/L, platelet count ≥100 × 10^9^/L (at screening and prior to chemotherapy), and Eastern Cooperative Oncology Group performance status ≤2 for patients ≥12 years of age.

Patients were excluded who had prior exposure to filgrastim, pegfilgrastim, lenograstim, or any other G-CSF in clinical study within 6 months before lipegfilgrastim administration; known hypersensitivity to filgrastim, pegfilgrastim, lenograstim, or any other G-CSF in clinical development; history of congenital neutropenia or cyclic neutropenia; previous bone marrow or stem cell transplantation or radiation to ≥25% of bone marrow for any reason, or any therapeutic radiation within 3 weeks before lipegfilgrastim administration; ongoing active infection or infectious disease within 2 weeks before lipegfilgrastim administration; or any illness or condition deemed by the study investigator to affect the safety of the patient or the evaluation of any endpoint.

### Treatment

Lipegfilgrastim (10 mg/mL) was administered at 100 µg/kg by a single subcutaneous injection 24 h (±3 h) after the last dose of chemotherapy was completed in week 1 of the regimen. The 100-µg/kg dose approximated the 6-mg dose used previously in adults on a weight-adjusted basis, and the maximum dose in this study was 6 mg. The abdomen was the preferred location for injection. Lipegfilgrastim was administered on day 4 of VIDE; day 3 of VDC/IE or IVA; and day 2, 3, 4, or 6 of VAC (depending on the individually chosen VAC regimen). All patients received the planned lipegfilgrastim dose. Administration of commercially available G-CSFs was not permitted during the study treatment period. Other supportive care was provided according to local standards in a manner consistent with the protocol.

### PK/PD and clinical assessments

In accordance with European Union guidelines, 30–35 mL total blood volume was drawn from the youngest group, and a maximum of 38 mL was drawn from the two older groups for the screening and treatment periods (4 weeks). Blood samples for PK analyses were obtained at baseline and at 3, 8, 24, 30, 48, 72, 96, 144, and 240 h postdose for the two older groups and up to 144 h for the youngest group. PK assessments were conducted on an inpatient or outpatient basis at the discretion of the investigator. The following PK parameters were estimated for each patient using noncompartmental analysis of the serum concentration–time data: *C*_max_ (maximum observed serum concentration), *t*_max_ (time to reach observed *C*_max_), AUC_0−*t*_ (area under the serum concentration–time curve from time 0 to the last measurable concentration, estimated using the linear trapezoidal rule method), AUC_0−inf_ (AUC from time 0 to infinity), *t*_1/2_ (terminal elimination half-life, estimated by linear regression), MRT (mean residence time), CL/F (apparent clearance), and V_z_/F (apparent volume of distribution).

Blood samples for PD assessments (ANC and CD34+ count) were collected before chemotherapy on day 1, before lipegfilgrastim administration, and on days 2, 4, 5, 6, 7, 8, 9, and 10 after lipegfilgrastim administration (except for patients in the youngest group weighing between ≥12.5 and <15 kg). Analysis of PD samples was performed at a central laboratory. PD assessments included ANC nadir, time to ANC nadir, time to ANC recovery from nadir to ≥1 × 10^9^/L and to ≥2 × 10^9^/L, and time to ANC recovery from first chemotherapy to ≥1 × 10^9^/L and to ≥2 × 10^9^/L. For CD34+ cells, area over baseline effect curve, AUC, maximum observed CD34+ count, and time to maximum observed CD34+ count were estimated.

The primary efficacy variable was the incidence of per protocol febrile neutropenia occurring in cycle 1, defined by an axillary or external ear temperature >38.3 °C or two consecutive readings of >37.8 °C at least 2 h apart, and ANC <0.5 × 10^9^/L. ANC and vital signs, including body temperature, were measured at baseline and throughout treatment. Incidence and duration of severe (ANC <0.5 × 10^9^/L) and very severe (ANC <0.1 × 10^9^/L) neutropenia were secondary measures of efficacy.

Adverse events (AEs) were assessed at screening and throughout the 3-week study period. Safety assessment comprised reported AEs, clinical laboratory test results, vital signs, 12-lead electrocardiography findings, physical examination findings, results of spleen sonography, and local tolerability at the injection site. The severity of each AE was recorded as either mild (easily tolerated), moderate (sufficiently discomforting to interfere with daily activity), or severe (preventing normal daily activities). A serious AE (SAE) was defined as one that resulted in death, was life threatening, resulted in persistent or significant incapacity, or necessitated medical intervention to prevent death or incapacity. The relationship between an AE and the study drug was classified as either “no reasonable possibility” (defined as AEs that were clearly due to extraneous causes such as disease or environment, or were considered unrelated to study drug after medical review at the time of evaluation) or “reasonable possibility” (defined as AEs for which a connection with the study drug could not be ruled out with certainty or there was a high degree of certainty related to the AE and the study drug).

### Statistical analysis

Analyses of PK, PD parameters (ANC and CD34+ counts), and investigator-assessed febrile neutropenia were conducted in the full analysis set, comprising all enrolled patients who received lipegfilgrastim and for whom at least one PK parameter could be derived. For all variables, only observed data from patients were used; missing data were not estimated. PK parameters and CD34+ analyses were performed using WinNonlin version 6.1 or later. Data points selected for the calculation of the terminal slope for each individual profile were used to perform PK analysis according to these criteria: The relative R^2^ of the line on semi-logarithmic plots is >0.8, at least three points were selected (not including *t*_max_), and the time interval used for determining the slope was at least two times the calculated *t*_1/2_. Drug concentrations less than the lower limit of quantification (LLOQ) were set to 0.5 × LLOQ before data analysis. For PD assessments, missing ANC values during cycle 1 were estimated using linear interpolation, which was performed only within the interval between the first and last available ANC measurements for cycle 1. Only patients with at least three ANC measurements during cycle 1 were considered evaluable.

Assessment of laboratory-defined efficacy (febrile neutropenia and severe/very severe neutropenia) per protocol definitions was conducted in the per protocol set, which excluded patients with any violation of inclusion/exclusion criteria, who had taken prohibited concomitant medications, who met withdrawal criteria but had not withdrawn, who had received incorrect doses of the study drug, or who missed assessments that might impact the study results. Safety and tolerability were assessed for all patients who received the study drug. Descriptive statistics were used for continuous and categorical variables and were provided for observed data only by stratified age group and by chemotherapy. Arithmetic and geometric means, standard deviations, minimum, maximum, median, and coefficients of variation were calculated for PK/PD parameters.

## Results

### Patients

Twenty-three pediatric patients with Ewing family of tumors or rhabdomyosarcoma scheduled to receive chemotherapy were screened for eligibility; 21 patients at 11 study centers in five countries (Czech Republic, Hungary, Poland, Russia, and Ukraine) met all inclusion criteria and were enrolled. All enrolled patients received study treatment and comprised the full analysis set. One patient in the youngest group was excluded from efficacy analysis because of missing PD data; therefore, 20 patients comprised the per protocol set (Figure S1).

Rhabdomyosarcoma was the primary cancer type in the youngest group, with embryonal rhabdomyosarcoma as the predominant subtype. Ewing sarcoma was most common in the two older groups and consisted primarily of Ewing tumor of bone (Table 1, Table S2). IVA was the planned chemotherapy regimen for most patients in the youngest group, and patients in the two older groups were predominantly treated with VIDE (Table 1).

**Table 1:** Patient demographics and baseline characteristics (full analysis set)

	Patients			
	2 to <6 years *n* = 7	6 to <12 years *n* = 7	12 to <18 years *n* = 7	Total *n* = 21
Median age (range), years	3 (2–5)	10 (7–11)	13 (13–16)	10 (2–16)
Sex, *n* (%)				
Male	5 (71)	3 (43)	4 (57)	12 (57)
Female	2 (29)	4 (57)	3 (43)	9 (43)
Median weight (range), kg	19.3 (12.8–20)	41.8 (23–44.2)	43.9 (24–63)	32 (12.8–63)
Race, *n* (%)				
White	7 (100)	7 (100)	7 (100)	21 (100)
Cancer type, *n* (%)				
Ewing family of tumors	1 (14)	5 (71)	6 (86)	12 (57)
Rhabdomyosarcoma	6 (86)	2 (29)	1 (14)	9 (43)
Median time from diagnosis (range), mo	0.3 (0.2–1.8)	0.1 (0.1–0.4)	0.2 (0–0.4)	0.3 (0–1.8)
Prior surgery, *n* (%)	7 (100)	5 (71)	5 (71)	17 (81)
Prior radiation, *n* (%)	0	0	0	0
Chemotherapy planned, *n* (%)				
IVA	5 (71)	0	0	5 (24)
VAC	1 (14)	2 (29)	1 (14)	4 (19)
VIDE	1 (14)	5 (71)	6 (86)	12 (57)

*IVA* ifosfamide+vincristine+actinomycin D; *VAC* vincristine+actinomycin D+cyclophosphamide; *VIDE* vincristine+ifosfamide+doxorubicin+etoposide

### Pharmacokinetic and pharmacodynamic results

**Fig. 1:**
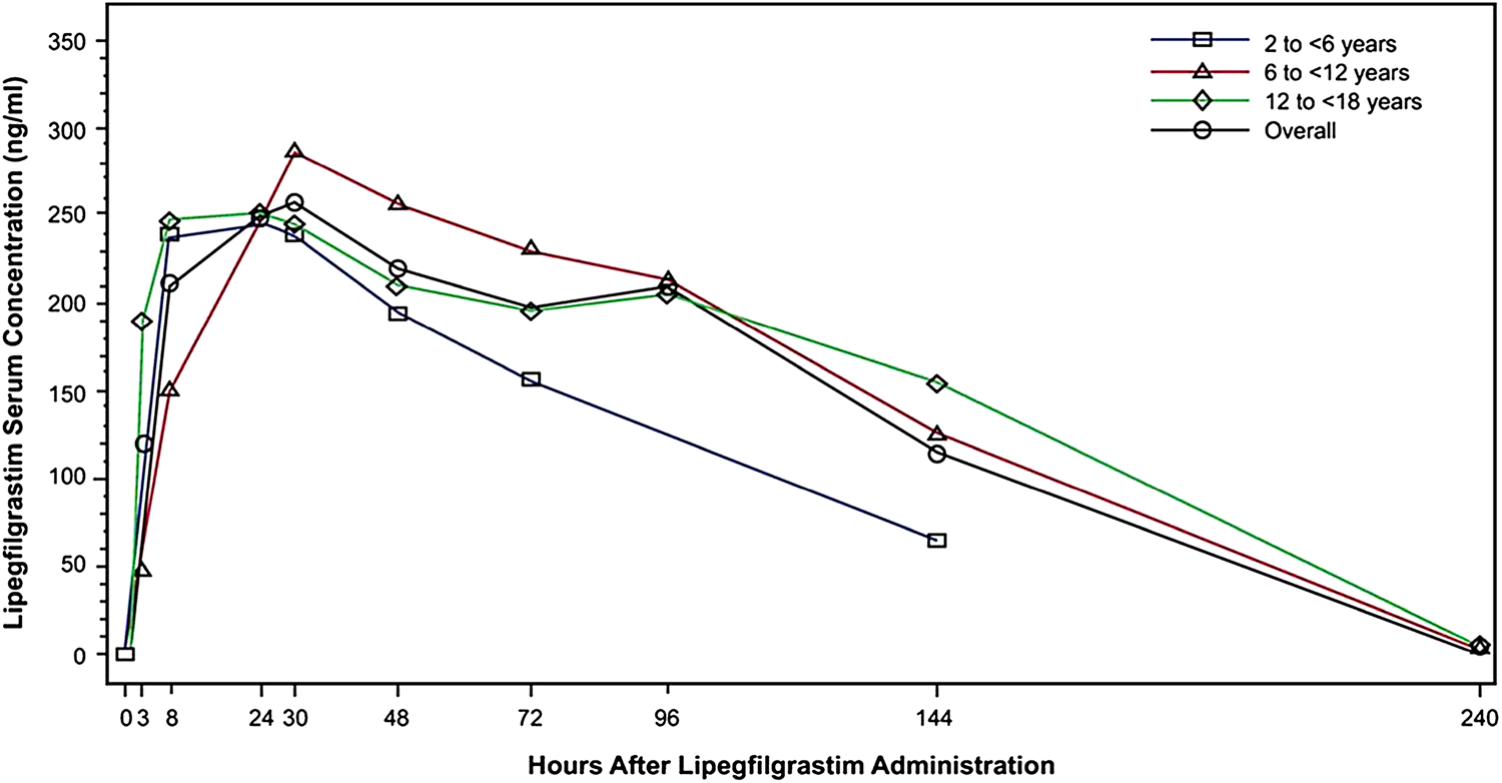
Serum concentration versus time plot following a single subcutaneous injection of lipegfilgrastim by age group (full analysis set). *Note* figure is in linear scale

Subcutaneous injection of lipegfilgrastim 100 µg/kg approximately 24 h after the last dose of chemotherapy in week 1 of the chemotherapy regimen resulted in mean (±SD) *C*_max_ values of 292 ± 178 ng/mL in the youngest age group, 303 ± 144 ng/mL in the mid-age group, and 341 ± 381 ng/mL in the oldest age group (Table 2). On average, lipegfilgrastim exposure levels were comparable across age groups, with concentrations of drug maintained over a prolonged period in each age group after a single injection (Fig. 1). *T*_max_ ranged between 45 and 82 h across all age groups (Table 2). The PK profile of at least one patient from each age group was shifted toward earlier *t*_max_ and higher *C*_max_ values compared with the remainder of the patients in each group, consistent with either more rapid systemic uptake of the drug or altered PD that affected the PK of the drug (Figures S2-S4). The effect was most apparent in one adolescent patient, resulting in the oldest age group having the highest variance in *C*_max_ (Table 2, Figure S4).

**Table 2:** Summary of pharmacokinetic parameters in the full analysis set

	Age group (years)		
	2 to <6 *n* = 7	6 to <12 *n* = 7	12 to <18 *n* = 7
*t*_max_, h	*n* = 7	*n* = 7	*n* = 7
Mean (SD)	50.3 (49.5)	45.4 (27.2)	82.2 (42.1)
Median (min, max)	23.9 (8.0, 144.0)	30.0 (29.9, 96.0)	95.8 (3.0, 142.0)
*C*_max_, ng/mL^a^	*n* = 7	*n* = 7	*n* = 7
Mean (SD)	292 (178)	303 (144)	341 (381)
Geometric mean (95% CI)	243 (128–460)	256 (128–510)	225 (90–560)
CV %	61.0	47.5	111.6
*t*_1/2_, h	*n* = 3	*n* = 7	*n* = 5
Mean (SD)	29.1 (14.3)	16.7 (3.1)	26.4 (12.6)
Median (min, max)	27.9 (15.4, 43.9)	17.4 (13.4, 22.4)	19.4 (16.1, 46.5)
AUC_0−t_, µg*h/mL^a^	*n* = 7	*n* = 7	*n* = 7
Geometric mean (95% CI)	17.7 (9.0–35.1)	30.0 (14.8–60.6)	27.4 (13.0–57.9)
CV %	65.7	47.2	60.7
AUC_0–inf_, µg*h/mL^a^	*n* = 3	*n* = 7	*n* = 5
Geometric mean (95% CI)	26.0 (4.5–149.6)	30.0 (14.8–60.6)	38.4 (20.4–72.0)
CV %	55.2	47.2	55.9
Vz/F, L^a^	*n* = 3	*n* = 7	*n* = 5
Geometric mean (95% CI)	2.72 (0.44–16.86)	2.86 (1.11–7.32)	4.08 (2.47–6.74)
CV %	66.4	154.7	49.9
CL/F, mL/h^a^	*n* = 3	*n* = 7	*n* = 5
Geometric mean (95% CI)	71 (17–300)	120 (53–270)	116 (49–276)
CV %	62.2	130.0	68.8
MRT^sc^, h^a^	*n* = 7	*n* = 7	*n* = 7
Geometric mean (95% CI)	49 (19–131)	79 (67–94)	90 (74–110)
CV %	42.1	16.7	14.6

*AUC* area under the serum concentration versus time curve; *CI* confidence interval; *CL*/*F* apparent clearance; *C*_max_ maximum serum concentration; *CV* coefficient of variation; *MRT* mean residence time following subcutaneous administration; *SD* standard deviation; *T*_1/2_ elimination half-life; *T*_max_ time to reach maximum serum concentration; *Vz/F* apparent volume of distribution during the terminal phase after non-intravenous administration

^a^All geometric means are rounded to the nearest whole number

Blood sampling stopped at 144 h for patients in the youngest group. Because fewer samples were collected in the elimination phase, the predefined conditions for calculating the terminal elimination rate constant and related parameters (i.e., *t*_½_, AUC_0−inf_, and CL/F) were not met for all patients, resulting in a complete set of PK parameters in only three of seven patients and five of seven patients in the youngest and oldest age group, respectively, compared with all seven patients in the mid-age group. Nevertheless, a meaningful PK comparison could be made among the groups with respect to lipegfilgrastim exposure parameters (*C*_max_ and AUC_0−*t*_), *t*_max_, and PK disposition parameters. Analysis of variance found no significant difference in the systemic exposure of lipegfilgrastim among the age groups (Table S3). Moreover, the geometric mean values and 95% confidence intervals for lipegfilgrastim MRT were consistent with a significantly prolonged PK disposition in the pediatric population (Table 2).

The mean ANC nadir was higher and the median time to ANC nadir from start of chemotherapy was longer in the youngest group (Table 3) than in the two older groups. The median time to ANC recovery from the start of chemotherapy was shorter in the youngest group than in the older groups. The median time to ANC recovery from nadir was the same in the two younger age groups and longest in the oldest group. Notably, patients aged 2 to <6 years received predominantly IVA chemotherapy, which is known to be associated with a less profound myelosuppressive effect than either VAC or VIDE. Patients treated with IVA demonstrated the highest mean ANC nadir and the shortest median ANC recovery time from the start of chemotherapy compared with those receiving either VAC or VIDE (Table 3).

**Table 3:** Summary of pharmacodynamic parameters (ANC and CD34+ count) by age and type of chemotherapy

	Age group (years)			Type of chemotherapy		
	2 to <6 *n* = 7	6 to <12 *n* = 7	12 to <18 *n* = 7	IVA *n* = 5	VAC *n* = 4	VIDE *n* = 12
ANC nadir, ×10^9^/L^a^	0.88 (0.76) *n* = 6	0.21 (0.35) *n* = 7	0.37 (0.77) *n* = 7	1.23 (0.71) *n* = 4	0.85 (0.93) *n* = 4	0.09 (0.08) *n* = 12
Time to ANC nadir from start of chemotherapy, days^b^	9 (7–17) *n* = 6	8 (6–12) *n* = 7	8 (8–10) *n* = 7	10 (8–17) *n* = 4	8 (6–12) *n* = 4	8 (7–10) *n* = 12
Time to ANC nadir from start of lipegfilgrastim, days^b^	6.5 (6–15) *n* = 6	5 (4–9) *n* = 7	5 (4–6) *n* = 7	8 (6–15) *n* = 4	6 (5–9) *n* = 4	5 (4–6) *n* = 12
Time to ANC ≥2 × 10^9^/L from start of chemotherapy, days^b^	11 (0–13) *n* = 4	12 (11–13) *n* = 7	12 (0–15) *n* = 7	6.5 (0–13) *n* = 2	11.5 (0–13) *n* = 4	12 (11–15) *n* = 12
Time to ANC ≥2 × 10^9^/L from ANC nadir, days^b^	3 (1–5) *n* = 4	3 (1–6) *n* = 7	4 (1–5) *n* = 7	3 (1–5) *n* = 2	2.5 (1–6) *n* = 4	3.5 (2–5) *n* = 12
ANC AUC, days ×10^9^/L^c^	65 (39–107) *n* = 6	60 (36–102) *n* = 7	81 (35–184) *n* = 7	79 (43–147) *n* = 4	61 (43–87) *n* = 4	67 (40–113) *n* = 12
Maximum CD34+ , cells/µL^a^	96.33 (66.07) *n* = 6	130.35 (123.19) *n* = 7	151.75 (122.87) *n* = 7	95.38 (71.34) *n* = 4	42.12 (32.04) *n* = 4	166.89 (114.10) *n* = 12
Time to CD34+ maximum from start of chemotherapy, days^b^	9.5 (7–13) *n* = 6	13 (7–15) *n* = 7	15 (10–19) *n* = 7	8 (7–11) *n* = 4	9.5 (7–13) *n* = 4	13.5 (11–19) *n* = 12
Time to CD34+ maximum from start of lipegfilgrastim, days^b^	7 (5–12) *n* = 6	9 (6–12) *n* = 7	12 (7–15) *n* = 7	6 (5–9) *n* = 4	6.5 (6–12) *n* = 4	10 (8–15) *n* = 12
Area over baseline effect curve for CD34+ , days*cells/µL^a^	356.09 (304.73) *n* = 6	466.32 (610.14) *n* = 7	688.25 (618.28) *n* = 7	306.66 (296.67) *n* = 4	116.32 (75.01) *n* = 4	710.55 (589.34) *n* = 12
CD34+ AUC, days*cells/µL^a^	402.20 (330.54) *n* = 6	518.42 (628.69) *n* = 7	705.13 (645.91) *n* = 7	374.01 (348.46) *n* = 4	125.32 (74.41) *n* = 4	748.40 (605.20) *n* = 12

All geometric means were rounded to the nearest whole number

*ANC* absolute neutrophil count; *AUC* area under the serum concentration–time curve; *IVA* ifosfamide+vincristine+actinomycin D; *VAC* vincristine+actinomycin D+cyclophosphamide; *VIDE* vincristine+ifosfamide+doxorubicin+etoposide

^a^Mean values and (standard deviation)

^b^Median (range)

^c^Geometric mean values and 95% confidence interval

The maximum CD34+ count was lowest and time to CD34+ maximum was shortest in the youngest group of patients (Table 3). Similar trends were observed for the mean area over baseline effect curve and geometric mean CD34+ AUC. Differences in median time to maximum CD34+ observed between age groups corresponded with chemotherapy; IVA was associated with the shortest time and VIDE with the longest.

### Efficacy

Treatment compliance, calculated as 100× body weight-adjusted dose/target dose, was approximately 100% for all patients. The median body weight-adjusted dose administered was 100 µg/kg for all three groups. Eight (38%) of the 21 patients in the full analysis set experienced febrile neutropenia by investigator assessment; in contrast, 4 of 20 patients (20%) in the per protocol set had laboratory-defined febrile neutropenia. When patients were stratified by age, the incidence of febrile neutropenia was highest in the oldest group by investigator- and laboratory-assessed definitions (Figure S5).

Stratification by type of chemotherapy showed that all 12 patients who received VIDE treatment experienced severe neutropenia regardless of age (Figure S6). In contrast, none of the four patients who received IVA and two of the four patients who received VAC experienced severe neutropenia. The duration of severe neutropenia was longest in the VIDE group (median 3.5 days) compared with the VAC (median 0.5 days) and IVA groups (none).

### Safety

Enrolled patients in all age groups each received a single dose of lipegfilgrastim; two patients received doses that exceeded 6 mg but were in proportion to recorded body weight. The mean absolute doses were 1.76, 3.68, and 4.58 mg for the 2- to <6-year, 6- to <12-year, and 12- to <18-year age groups, respectively.

Each of the 21 patients experienced at least one AE, with a total of 142 events reported. The most common AEs occurring in more than 15% of patients in any age group were neutropenia, febrile neutropenia, thrombocytopenia, leukopenia, anemia, abdominal pain, constipation, nausea, and stomatitis (Table 4). The highest frequency of hematologic AEs, including febrile neutropenia, thrombocytopenia, and leukopenia, was reported in the oldest group.

**Table 4:** Overall adverse events occurring in at least two patients, by age group

*n* (%)	2 to <6 years *n* = 7	6 to <12 years *n* = 7	12 to <18 years *n* = 7	Total *n* = 21
Hematologic adverse events				
Neutropenia	4 (57.1)	3 (42.9)	4 (57.1)	11 (52.4)
Febrile neutropenia	1 (14.3)	2 (28.6)	5 (71.4)	8 (38.1)
Thrombocytopenia	3 (42.9)	1 (14.3)	4 (57.1)	8 (38.1)
Leukopenia	2 (28.6)	1 (14.3)	4 (57.1)	7 (33.3)
Anemia	3 (42.9)	0	3 (42.9)	6 (28.6)
Nonhematologic adverse events				
Abdominal pain	0	3 (42.9)	1 (14.3)	4 (19.0)
Constipation	1 (14.3)	2 (28.6)	1 (14.3)	4 (19.0)
Nausea	0	3 (42.9)	1 (14.3)	4 (19.0)
Stomatitis	2 (28.6)	0	2 (28.6)	4 (19.0)
Aspartate aminotransferase increase	1 (14.3)	1 (14.3)	1 (14.3)	3 (14.3)
Decreased appetite	0	2 (28.6)	1 (14.3)	3 (14.3)
Vomiting	1 (14.3)	0	1 (14.3)	2 (9.5)
Abdominal pain upper	0	2 (28.6)	0	2 (9.5)
Alanine aminotransferase increase	0	1 (14.3)	1 (14.3)	2 (9.5)
Neutrophil count decrease	1 (14.3)	1 (14.3)	0	2 (9.5)
Back pain	0	2 (28.6)	0	2 (9.5)

Includes treatment-emergent and treatment-related events

Treatment-related AEs (all mild in severity) were experienced by one patient in the 2- to <6-year age group (increased neutrophil count) and one in the 6- to <12-year age group (back and bone pain).

Severe AEs were experienced by 57% of patients; the most common (occurring in >15% of patients in any age group) were febrile neutropenia, leukopenia, and neutropenia. The incidence of severe hematologic AEs was highest in the oldest group (Table S4). None of the severe AEs reported were classified as treatment related. No significant changes in serum chemistries were observed. Similarly, no clinically significant changes in spleen size were observed via sonography.

No deaths were reported following lipegfilgrastim administration. A total of five SAEs, all of which necessitated hospitalization, were reported in three patients treated with VIDE. One patient (age 10 years) had febrile neutropenia and very severe neutropenia, which resolved after 2 and 4 days, respectively. A second patient (age 13 years) had severe neutropenia and febrile neutropenia, which resolved after 4 days, and a third (age 14 years) had febrile neutropenia lasting 3 days. All SAEs were managed with antibiotics and ibuprofen. None of the SAEs were considered related to lipegfilgrastim.

## Discussion

Treatment with dose-intensive chemotherapy for children with sarcoma has been limited by myelosuppressive side effects and associated infectious complications. Filgrastim is the only G-CSF approved in Europe for children as a daily injection, which may be uncomfortable for children. The long-acting G-CSF pegfilgrastim has been approved in Europe for adults but not for children. Few prospective studies have evaluated the PK of available G-CSF regimens in children with sarcoma [9, 10].

This is the first study to report clinical pharmacology data for the long-acting G-CSF lipegfilgrastim in a pediatric population. This study was designed to examine the PK, as well as the PD, safety, and efficacy, of a single subcutaneous dose of lipegfilgrastim among pediatric patients. Overall, the *C*_max_, AUC_0−*t*_, and MRT results of this study support the use of a 100-μg/kg dose of lipegfilgrastim for patients 2 to <18 years of age. Based on lipegfilgrastim *C*_max_, in particular, the use of a body weight-adjusted dose resulted in early lipegfilgrastim exposure levels that were comparable across age groups. Moreover, the peak exposure levels of lipegfilgrastim in each pediatric age group were comparable to those reported in various groups of adult cancer patients receiving chemotherapy [13]. The observed differences in *t*_max_ with lipegfilgrastim may be attributed to variability between individual patients. Analysis of variance in PK parameters of interest detected no differences across age groups, although missing data from patients in the youngest and oldest groups limit meaningful interpretation of some PK parameters.

Some PK values in this study are comparable to those observed in pegfilgrastim-supported pediatric patients. The average *C*_max_ values of lipegfilgrastim correspond with those observed in pegfilgrastim-treated patients [10]. The median *t*_max_ values observed in this study are comparable with those in pediatric patients after pegfilgrastim administration [9, 10]. Similarly, the mean *t*_1/2_ values of lipegfilgrastim are higher in pediatric patients <6 years of age than are those in older children [10]. These trends suggest that lipegfilgrastim and pegfilgrastim, as expected from their molecular structures, may share some PK properties.

**Fig. 2:**
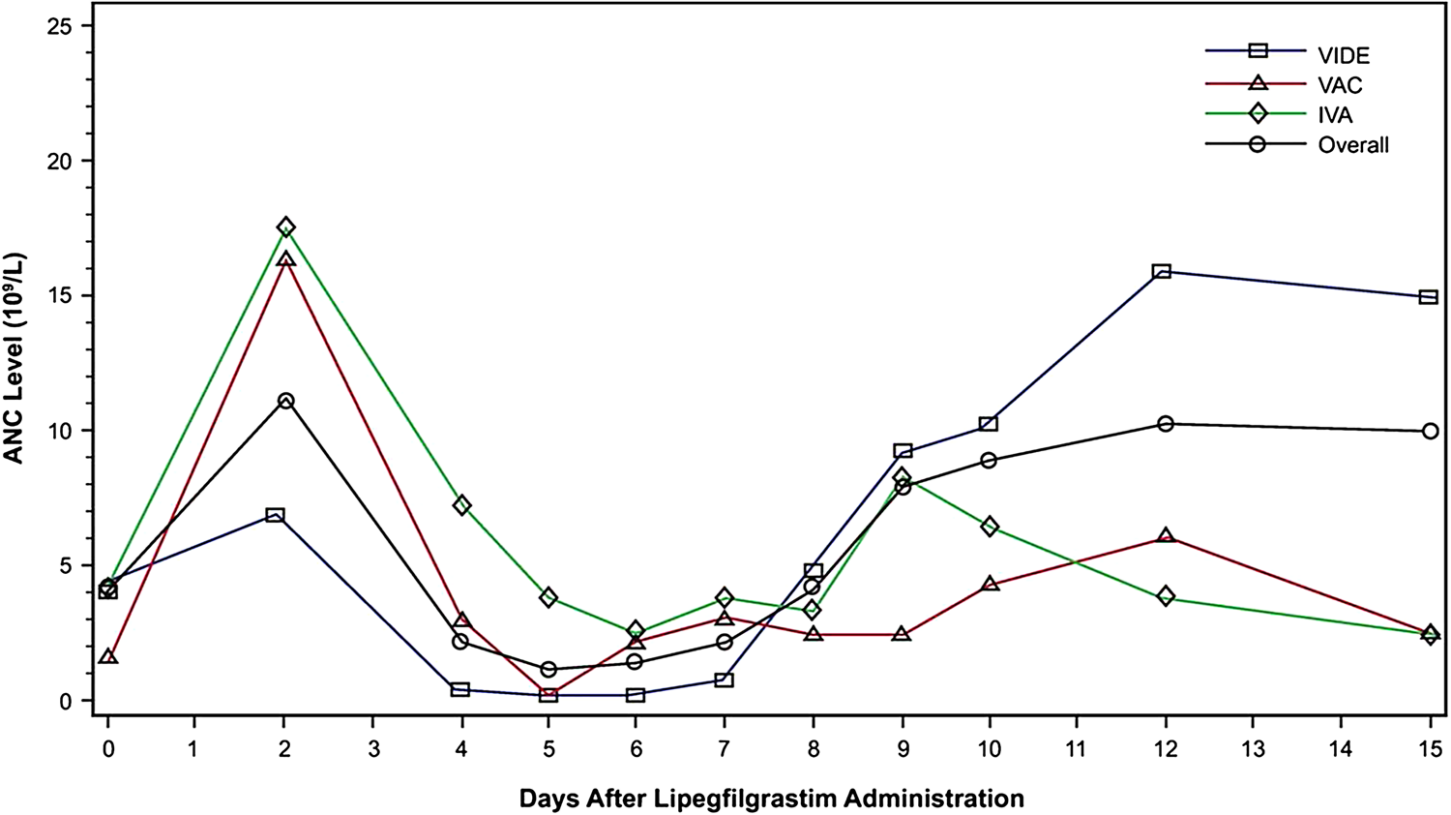
Absolute neutrophil count by type of chemotherapy (full analysis set). *Note* figure is in linear scale. *ANC* absolute neutrophil count; *IVA* ifosfamide/vincristine/actinomycin D; *VAC* vincristine/actinomycin D/cyclophosphamide; *VIDE* vincristine/ifosfamide/doxorubicin/etoposide

The mean ANC nadir was highest for the youngest patients in the study. Differences in ANC counts were associated with the types of chemotherapy administered (Fig. 2). Patients in the youngest group primarily received IVA chemotherapy, which is known to produce less myelosuppression than either VAC or VIDE. Among the three chemotherapy regimens used in this study, VIDE was the most myelosuppressive, with the lowest ANC nadir; however, lipegfilgrastim has been shown to stimulate the highest recovery of neutrophils in patients who received VIDE, along with the highest CD34+ level in the recovery period (Table 3), compared with the other chemotherapy regimens in this study.

The majority of investigator-reported AEs were related to chemotherapy and not to lipegfilgrastim. The only serious AEs were neutropenia and febrile neutropenia, which were reported in 3 of 21 patients (14.29%). Stratification by type of chemotherapy showed that VIDE treatment, which was predominantly received by patients 6 to <18 years of age, was associated with the highest incidence of laboratory-assessed febrile neutropenia (4/12 patients, or 33%; Figure S5), particularly among patients 12 to <18 years of age (3/6 patients, or 50%). These results suggest that there may be an association between the incidence of febrile neutropenia and the age of pediatric patients, with older patients experiencing a higher incidence. These combined results also raise the possibility of an association between febrile neutropenia and either VIDE or the underlying disease type, particularly among older pediatric patients, as VIDE was administered only to patients with Ewing family of tumors. Therefore, it is not possible to draw a clear conclusion regarding an association between febrile neutropenia and patient age, and further study is warranted.

The association between type of chemotherapy and incidence of febrile neutropenia aligns with the few published reports on the use of other G-CSFs in pediatric patients. In a study of pediatric patients with Ewing sarcoma treated with VIDE, Wendelin and colleagues reported febrile neutropenia in 78% and 56% of cycles in which pegfilgrastim and filgrastim were given, respectively [17]. Likewise, in a retrospective analysis of pediatric patients treated with pegfilgrastim, André and colleagues reported febrile neutropenia in 47%, 4%, and 33% of patients treated with VIDE, VAC, and vincristine/ifosfamide/dactinomycin/doxorubicin, respectively [7].

In a randomized phase 2 study of pediatric patients with sarcoma, 68% of those receiving pegfilgrastim and 83% of patients treated with filgrastim experienced febrile neutropenia [10]. Over the course of the present study, investigator-assessed febrile neutropenia was recorded in 38% of patients. Central laboratory findings differ, showing febrile neutropenia in 20% of patients. When patients were stratified by age, the incidence of febrile neutropenia was highest in the oldest group according to both investigator-assessed (71.4%) and laboratory-assessed (42.9%) definitions.

The open-label, uncontrolled design of the present study limits the conclusions possible regarding the safety and efficacy of lipegfilgrastim in the pediatric population. The single injection of lipegfilgrastim prevents further interpretation of individual toxicity experiences or immunogenicity. A second, controlled study with a larger cohort of pediatric patients with Ewing family of tumors or rhabdomyosarcoma treated with IVA, VAC, or VIDE is planned to further investigate the efficacy and safety of lipegfilgrastim and filgrastim administered over multiple cycles. The primary efficacy endpoint of this study will be the duration of severe neutropenia in cycle 1 by treatment, age group, and baseline ANC value. Given the strong effect of chemotherapy observed in the present study, the type of chemotherapy will be added as a stratification variable.

In conclusion, PK data in this study support the use of a 100-μg/kg dose of lipegfilgrastim for patients 2 to <18 years of age. Lipegfilgrastim exposure levels were comparable across age groups, with concentrations maintained over a prolonged period after a single injection. Preliminary evidence suggests that lipegfilgrastim may be safe and well tolerated for at least 3 weeks following administration in the pediatric population studied. Severe AEs were associated with chemotherapy, particularly in patients receiving VIDE. Febrile neutropenia, either alone or in addition to neutropenia, accounted for all reported SAEs, which occurred in three patients who received VIDE chemotherapy. These PK and safety data support ongoing investigation of lipegfilgrastim in all three age groups.

## Electronic supplementary material

Below is the link to the electronic supplementary material.

Supplementary material 1 (DOCX 764 kb)
