# Supplementary material for: A pharmacokinetic study of lipegfilgrastim in children with Ewing family of tumors or rhabdomyosarcoma
Source: Cancer Chemother Pharmacol. 2016 Dec 16;79(1):155–64. doi: 10.1007/s00280-016-3216-2 (PMC5225185; doi:10.1007/s00280-016-3216-2)
Supplement: Supplementary file 1 — Supplementary material 1 (DOCX 764 kb) [file 280_2016_3216_MOESM1_ESM.docx]

**Supplementary Material (6 figures/4 tables)**

**Belogurova MB, et al.**

**A Pharmacokinetic Study of Lipegfilgrastim in Children With Ewing Family of Tumors or Rhabdomyosarcoma**

**Figure S1. Patient disposition.**

**
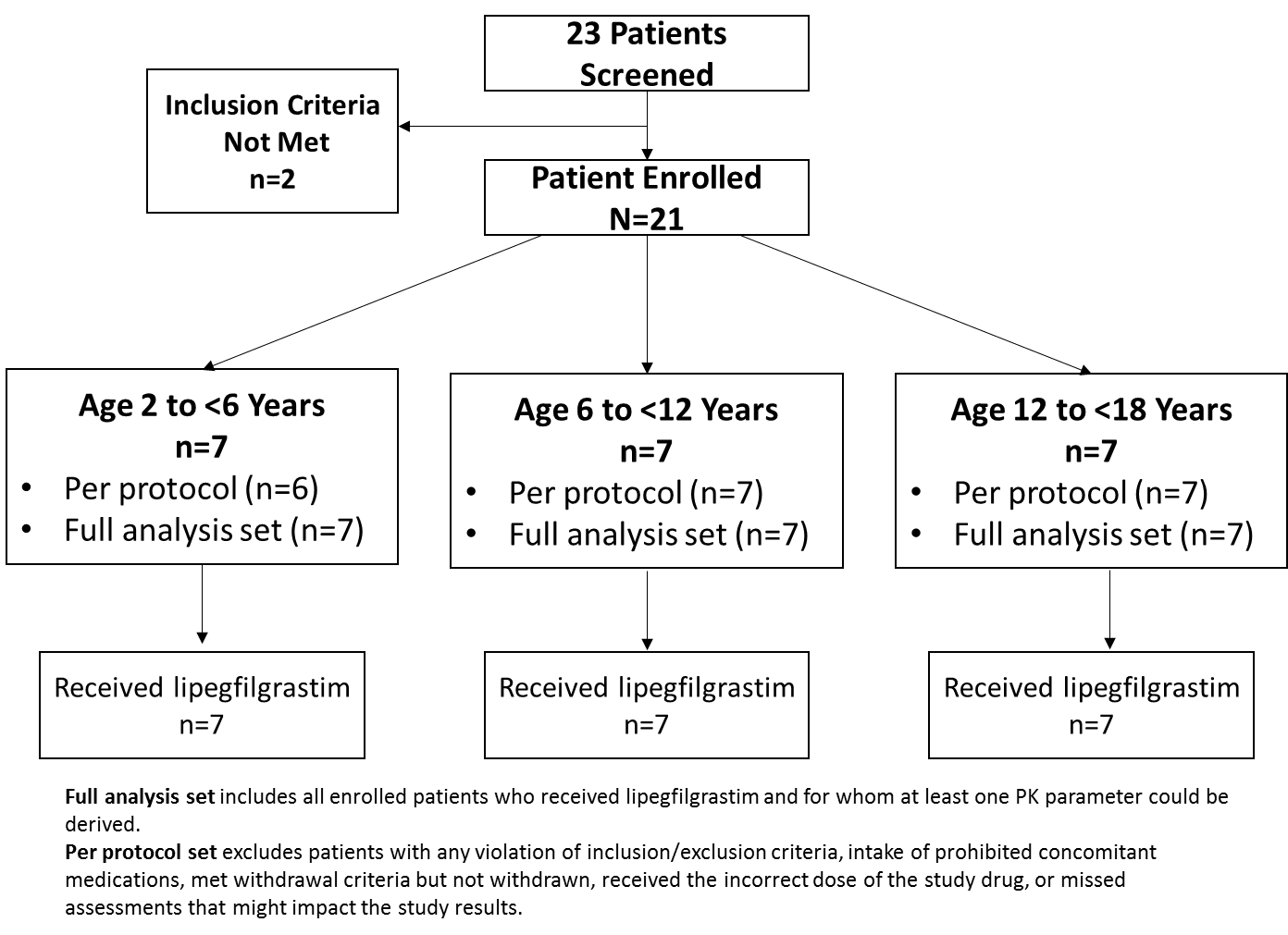
**

**Figure S2. Serum concentration versus time plot following a single subcutaneous injection of lipegfilgrastim by patient, age 2 to <6 years (full analysis set).** Note: Figure is in linear scale.


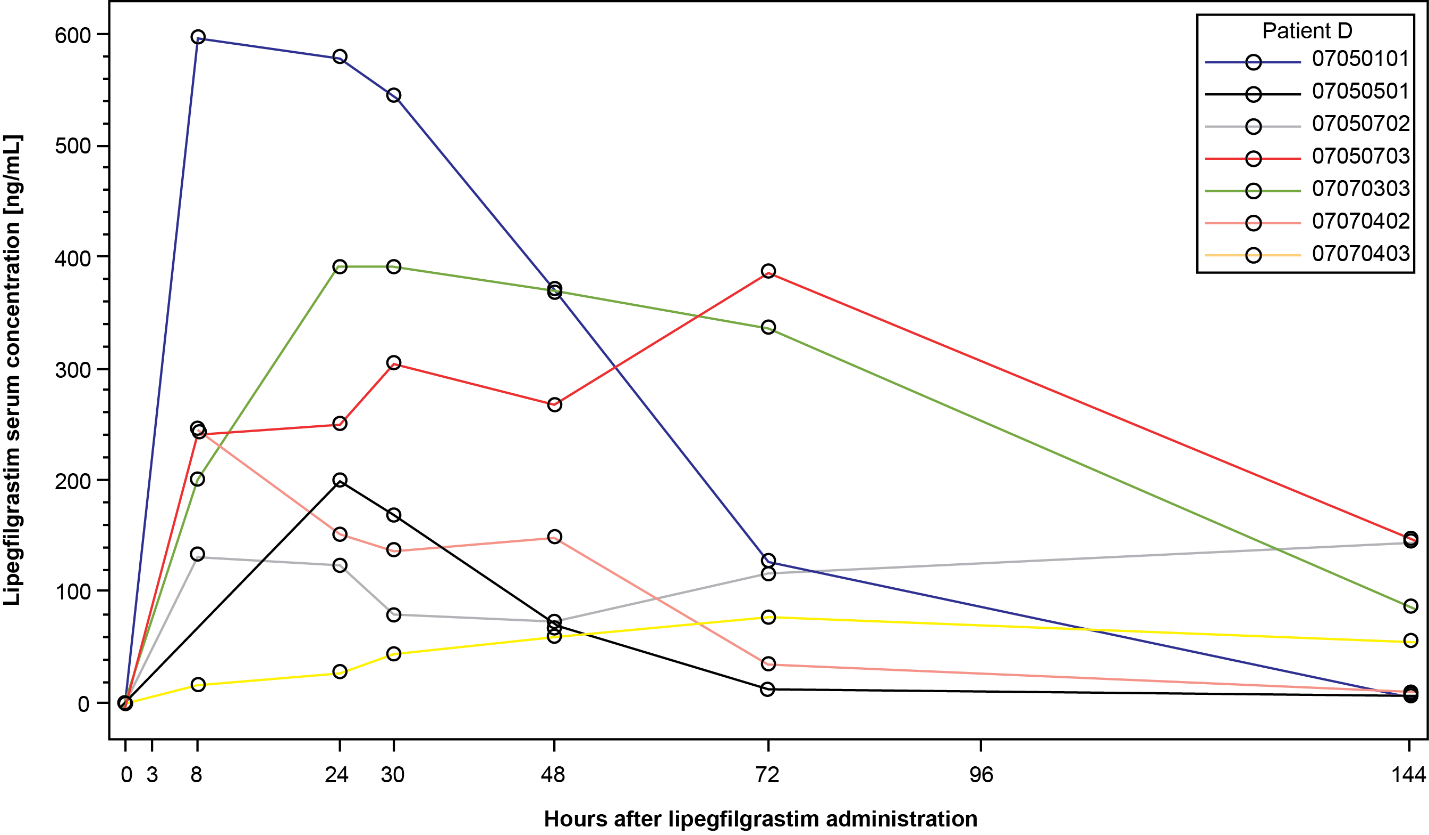


**Figure S3. Serum concentration versus time plot following a single subcutaneous injection of lipegfilgrastim by patient, age 6 to <12 years (full analysis set).** Note: Figure is in linear scale.


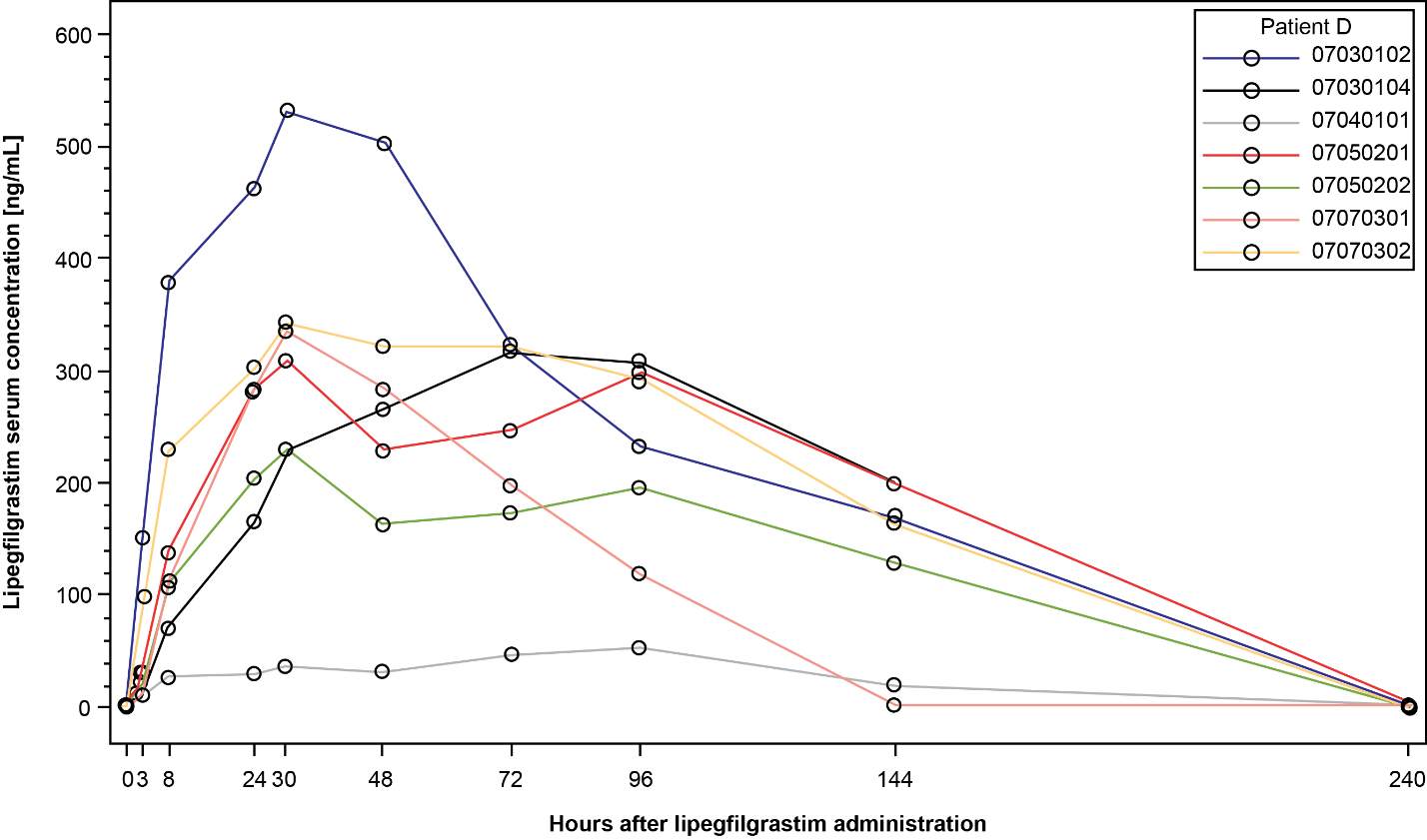


**Figure S4. Serum concentration versus time plot following a single subcutaneous injection of lipegfilgrastim by patient, age 12 to <18 years (full analysis set).** Note: Figure is in linear scale.


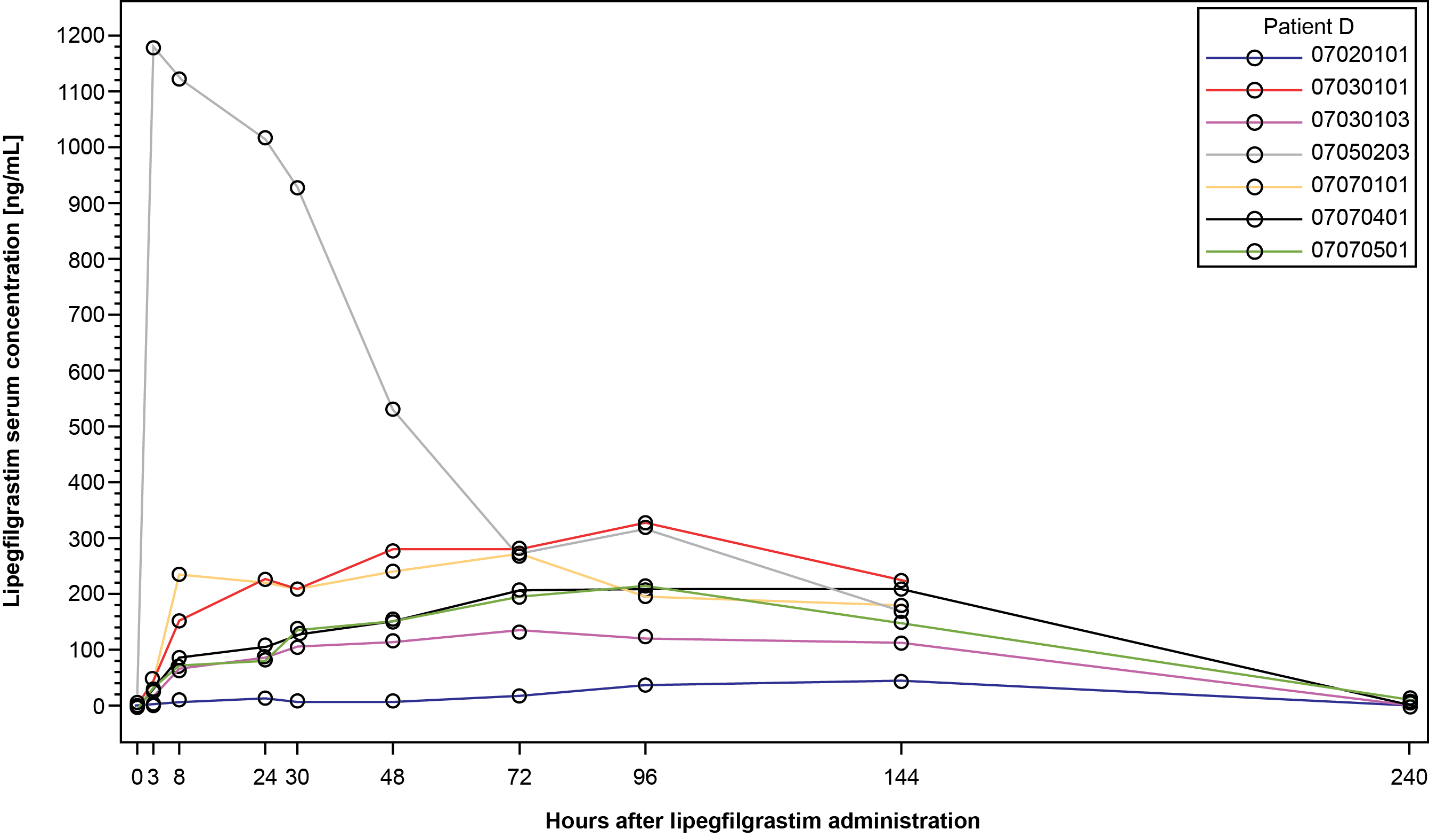


**Figure S5. Patients with febrile neutropenia in cycle 1 by age and chemotherapy regimen administered per investigator assessment in full analysis set or laboratory assessment in per protocol set.**


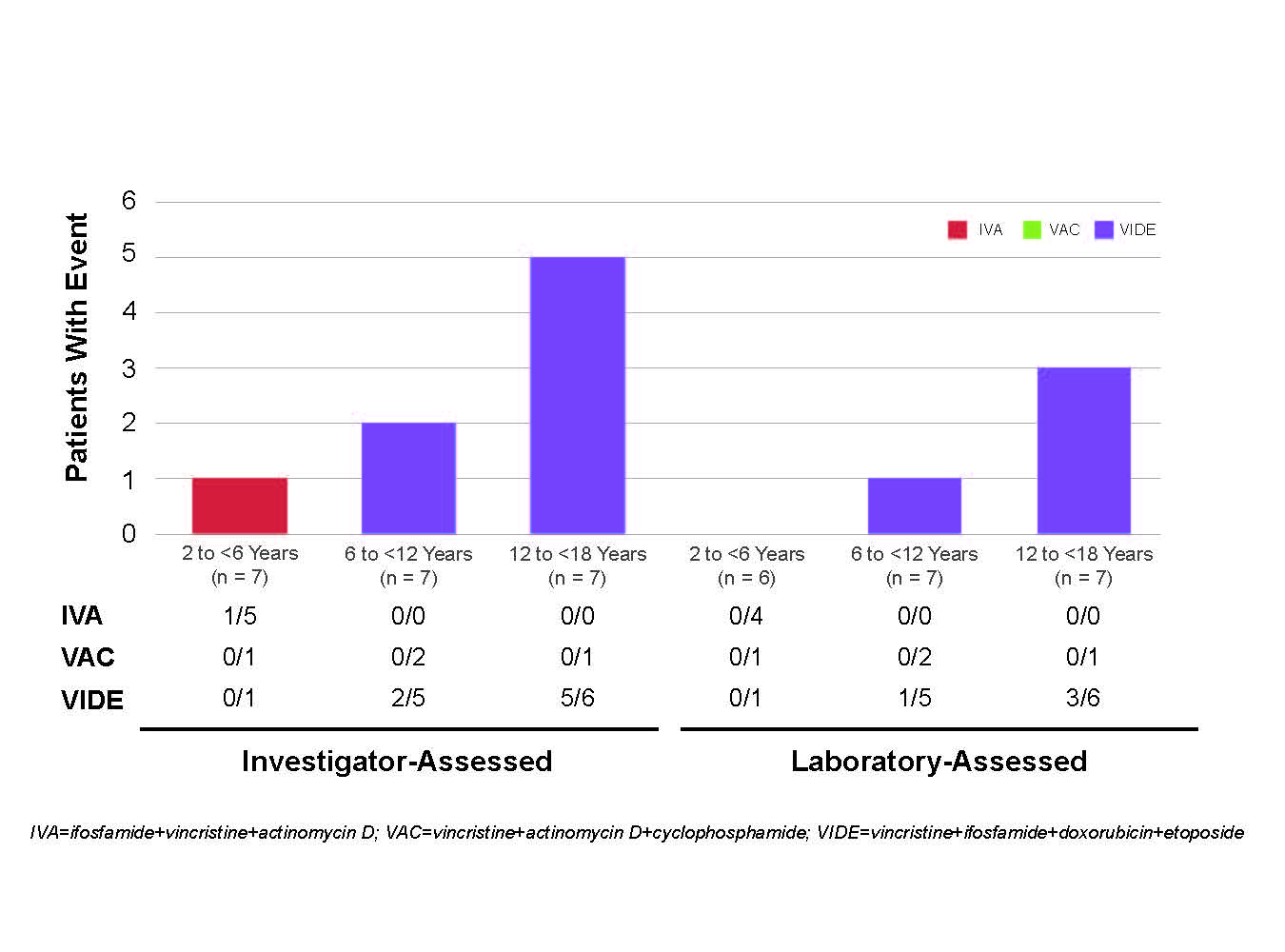


**Figure S6. Patients with severe or very severe neutropenia in cycle 1 by age and chemotherapy regimen administered.**


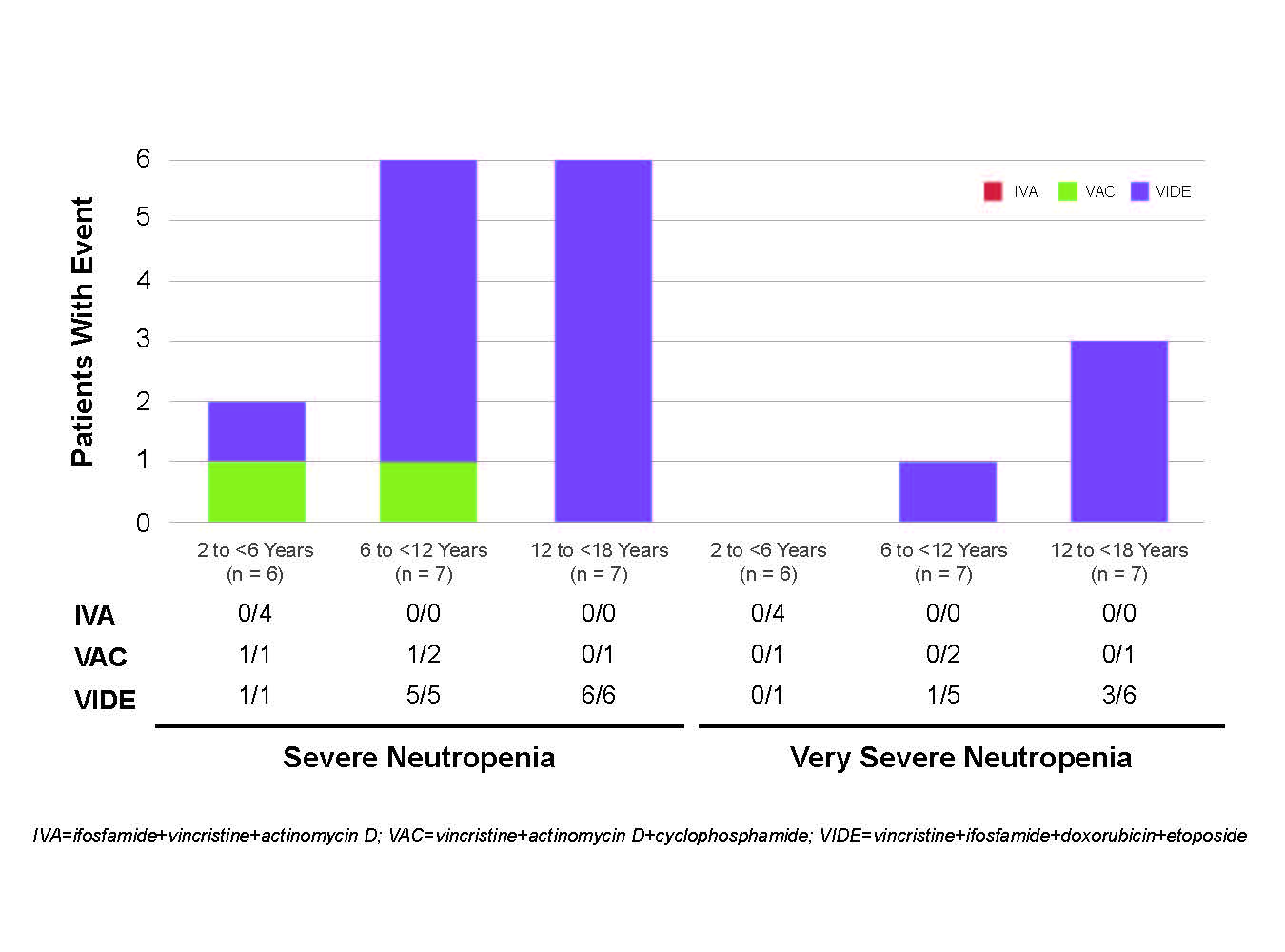


**Supplementary Tables**

**Table S1. Chemotherapy Regimens for Enrolled Patients**

|  | **Chemotherapy Regimen** | **Dose and Schedule** |
| --- | --- | --- |
| **Ewing family of tumors** | **VIDE** | Vincristine 1.5 mg/m² day 1  Ifosfamide 3.0 g/m² day 1, day 2, day 3  (plus MESNA at dosages per local standard of care for prophylaxis for bladder complications)  Doxorubicin 20 mg/m² day 1, day 2, day 3  Etoposide 150 mg/m² day 1, day 2, day 3 |
|  | **VDC/IE** | VDC cycles 1 and 3  Vincristine 2.0 mg/m² day 1, 8, and 15  Doxorubicin 75 mg/m² day 1  Cyclophosphamide 1200 mg/m²/day 1 and 2  (plus MESNA at dosages per local standard of care for prophylaxis for bladder complications)  IE cycles 2 and 4  Ifosfamide 1800 mg/m²/day x 5 days  Etoposide 100 mg/ m²/day x 5 days  (plus MESNA at dosages per local standard of care for prophylaxis for bladder complications)  Note: Patients receive only 1 cycle of chemotherapy during lipegfilgrastim treatment.  The patients who receive alternating VDC and IE chemotherapy must start with the VDC cycle 1 during the treatment period of the study and lipegfilgrastim on day 3. |
| **Rhabdomyosarcoma** | **VAC** | Vincristine 1.5 mg/m² day 1, 8, and 15; not to exceed 2 mg on one day  Actinomycin D 0.015 mg/kg/d IV push for 5 days or 1.5 mg/m² IV  push every 3 weeks  Cyclophosphamide 1.2-2.2 g/m²/day IV for 1-3 days starting on day 1  (plus MESNA at dosages per local standard of care for prophylaxis for bladder complications) |
|  | **VDC/IE** | Cycles administered as for Ewing’s sarcoma |
|  | **IVA** | Ifosfamide 3.0 g/m^2^ on day 1 and day 2  Vincristine 1.5 mg/m^2^ on day 1, day 8, and day 15  Actinomycin D 1.5 mg/m^2^ on day 1  (plus MESNA at dosages per local standard of care for prophylaxis for bladder complications) |

IE, ifosfamide/etoposide; IVA, ifosfamide/vincristine/actinomycin D; MESNA, sodium 2-mercaptoethane sulfonate; VAC, vincristine/actinomycin D/cyclophosphamide; VIDE, vincristine/ifosfamide/doxorubicin/etoposide; VDC, vincristine, doxorubicin, and cyclophosphamide.

**Table S2. Tumor Subtypes by Age Group**

| **n (%)** | **2 to <6 years**  **n=7** | **6 to <12 years**  **n=7** | **12 to <18 years**  **n=7** |
| --- | --- | --- | --- |
| **Ewing family of tumors** | 1 (14.3) | 5 (71.4) | 6 (85.7) |
| Ewing tumor of bone | 1 (14.3) | 4 (57.1) | 5 (71.4) |
| Extraosseous Ewing tumors | 0 | 0 | 0 |
| Peripheral primitive neuroectodermal tumor | 0 | 1 (14.3) | 1(14.3) |
| **Rhabdomyosarcoma** | 6 (85.7) | 2 (28.6) | 1 (14.3) |
| Embryonal rhabdomyosarcoma | 4 (57.1) | 1 (14.3) | 0 |
| Botryoid rhabdomyosarcoma | 0 | 0 | 0 |
| Spindle cell rhabdomyosarcoma | 0 | 0 | 0 |
| Alveolar rhabdomyosarcoma | 2 (28.6) | 1 (14.3) | 1 (14.3) |
| Pleomorphic and undifferentiated rhabdomyosarcoma | 0 | 0 | 0 |

**Table S3. Analysis of Variance in Pharmacokinetic Parameters (Full Analysis Set)**

|  | **2 to <6 years**  **n=7** | **6 to <12 years**  **n=7** | **12 to <18 years**  **n=7** | ***P* value^a^ for age group effect** |
| --- | --- | --- | --- | --- |
| **AUC_0-t_, µg*h/mL** | n=7 | n=7 | n=7 |  |
| LS mean, estimate (SE) | 17.7 (0.001) | 30.0 (0.001) | 27.4 (0.001) | 0.4130 |
| 95% CI | 9.6-32.7 | 16.2-55.2 | 14.9-50.5 |  |
| **C_max_, ng/mL** | n=7 | n=7 | n=7 |  |
| LS mean, estimate (SE) | 243.1 (1.4) | 255.9 (1.4) | 224.9 (1.4) | 0.9569 |
| 95% CI | 126-465.4 | 133.6-489.9 | 117.5-430.6 |  |
| **Vz/F, L** | n=3 | n=7 | n=5 |  |
| LS mean, estimate (SE) | 2.72 (0.002) | 2.86 (0.002) | 4.08 (0.001) | 0.7125 |
| 95% CI | 0.98-7.58 | 1.46-5.58 | 1.84-9.01 |  |
| **CL/F, mL/h** | n=3 | n=7 | n=5 |  |
| LS mean, estimate (SE) | 70.8 (1.6) | 119.9 (1.3) | 115 (1.4) | 0.6038 |
| 95% CI | 26.7-188 | 63.2-227.2 | 54.4-247.1 |  |

^a^*P* value from analysis of variance model ln(parameter)=age_group.

AUC, area under the serum concentration vs time curve; CI, confidence interval; CL/F, apparent clearance; C_max_, maximum serum concentration; LS, least squares; SE, standard error; Vz/F, apparent volume of distribution during the terminal phase after non-intravenous administration

**Table S4. Moderate and Severe Treatment-Emergent Adverse Events by Age Group**

| **n (%)** | **2 to <6 years**  **n=7** | | **6 to <12 years**  **n=7** | | **12 to <18 years**  **n=7** | |
| --- | --- | --- | --- | --- | --- | --- |
|  | **Moderate** | **Severe** | **Moderate** | **Severe** | **Moderate** | **Severe** |
| Anemia | 0 | 0 | 0 | 0 | 1 (14.3) | 1 (14.3) |
| Febrile neutropenia | 0 | 1 (14.3) | 0 | 2 (28.6) | 3 (42.9) | 2 (28.6) |
| Leukopenia | 0 | 2 (28.6) | 0 | 1 (14.3) | 1 (14.3) | 3 (42.9) |
| Neutropenia | 0 | 4 (57.1) | 1 (14.3) | 2 (28.6) | 0 | 3 (42.9) |
| Thrombocytopenia | 2 (28.6) | 0 | 0 | 0 | 3 (42.9) | 0 |
| Blood pressure decreased | 0 | 0 | 0 | 0) | 0 | 1 (14.3) |
| Neutrophil count decreased | 1 (14.3) | 0 | 0 | 1 (14.3) | 0 | 0 |
| Decreased appetite | 0 | 0 | 2 (28.6) | 0 | 0 | 1 (14.3) |
| Abdominal pain | 0 | 0 | 1 (14.3) | 0 | 0 | 0 |
| Abdominal pain, upper | 0 | 0 | 1 (14.3) | 0 | 0 | 0 |
| Constipation | 0 | 0 | 1 (14.3) | 0 | 0 | 0 |
| Stomatitis | 0 | 0 | 0 | 0 | 1 (14.3) | 0 |
| Vomiting | 1 (14.3) | 0 | 0 | 0 | 1 (14.3) | 0 |
